# Cervical muscle morphometry and composition demonstrate prognostic value in degenerative cervical myelopathy outcomes

**DOI:** 10.3389/fneur.2023.1209475

**Published:** 2023-09-07

**Authors:** Neda Naghdi, James M. Elliott, Michael H. Weber, Michael G. Fehlings, Maryse Fortin

**Affiliations:** ^1^Department of Health, Kinesiology & Applied Physiology, Concordia University, Montreal, QC, Canada; ^2^The Kolling Institute, The University of Sydney, Sydney, NSW, Australia; ^3^The Northern Sydney Local Health District, Royal North Shore Hospital, St Leonards, NSW, Australia; ^4^Montreal General Hospital Site, Department of Orthopedic Surgery, McGill University Health Centre, Montreal, QC, Canada; ^5^Department of Neurosurgery and Spinal Program, University of Toronto, Toronto, ON, Canada; ^6^PERFORM Centre, Concordia University, Montreal, QC, Canada; ^7^Centre de Recherche Interdisciplinaire en Readaptation (CRIR), Montreal, QC, Canada

**Keywords:** cervical extensor muscles morphology, cervical extensor muscles composition, degenerative cervical myelopathy, magnetic resonance imaging, post-operative outcome

## Abstract

**Objectives:**

This study aimed to examine whether preoperative cervical muscle size, composition, and asymmetry from magnetic resonance imaging (MRI) can predict post-operative outcomes in patients with degenerative cervical myelopathy (DCM).

**Methods:**

A total of 171 patients with DCM were included. Relative total cross-sectional area (RCSA), functional CSA (fat-free area, FCSA), ratio of FCSA/CSA (fatty infiltration) and asymmetry of the multifidus (MF) and semispinalis cervicis (SCer) together (MF + SCer), and cervical muscle as a group (MF, SCer, semispinalis capitis, and splenius capitis) were obtained from T2-weighted axial MR images at the mid-disk, at the level of maximum cord compression and the level below. Univariate and multivariate linear regression analyses were used to assess the relationship between baseline cervical muscle measurements of interest with the modified Japanese Orthopedic Association (mJOA), Nurick Classification, Neck Disability Index (NDI), and SF-36 health survey at 6-month and 12-month post-surgery.

**Results:**

Lower RCSA of MF + SCer, less CSA MF + SCer asymmetry and greater FCSA/CSA for the cervical muscle group (e.g., less fatty infiltration), and younger age were significant predictors of higher mJOA scores (e.g., less disability) at 6-month and 12-month post-surgery (all *p* < 0.05). Greater CSA asymmetry in MF + SCer and lower FCSA/CSA (e.g., more fatty infiltration) for the cervical muscle group were significant predictors of higher Nurick scores (e.g., more disability) at 6-month and 12-month post-surgery (all *p* < 0.05). Lower FCSA MF + Scer asymmetry, lower FCSA/CSA asymmetry of the muscle group, and greater RCSA MF + SCer were significant predictors of higher NDI scores at 6-month and 12-month post-surgery. Finally, greater FCSA/CSA asymmetry of the MF + SCer, greater FCSA asymmetry of the muscle group, greater RCSA of the muscle group, and greater CSA asymmetry of MF + SCer were significant predictors of lower post-operative SF-36 scores at 6- and 12-month post-surgery.

**Conclusion:**

Our result suggested that cervical paraspinal muscle morphology, specifically greater asymmetry, and fatty infiltration may be important predictors of functional recovery and post-surgical outcomes in patients with DCM.

## 1. Introduction

Degenerative cervical myelopathy (DCM) is the most prevalent cause of spinal cord dysfunction in adults worldwide (1–5). This age-related disorder of the cervical spine is associated with a progressive narrowing of the spinal canal, leading to pain and neurological impairment (2). In accordance with the World Health Organization, the number of people aged 60 years and over is expected to increase from 11% in 2010 to 22% in 2050 (2). Accordingly, health professionals globally will be expected to address a growing number of spinal disorders associated with advanced aging, particularly DCM (2, 4). Muscle hypotrophy occurs naturally and is proportional to aging, a possible confounding factor when assessing predictors of outcome (6). Common anatomical features of the aging spine include the degeneration of facet joints, intervertebral disks and/or vertebral bodies, hypertrophy of the ligamentum flavum, and ossification of the longitudinal ligament (OPLL) (5). While not mutually exclusive, all or any of these features can contribute to persistent compression of the spinal cord overtime (4, 7). Due to mechanical compression of the neural components, roughly 40% of individuals with features of and clinical indications for spinal degeneration will develop symptoms of neurological impairment (1, 2). The clinical presentation of DCM includes, but is not limited to, neck stiffness, gait impairment, numbness of the hands, and even tetraplegia (1, 8). While decompressive surgery is considered a practical option for patients with progressive DCM (1), nearly 40% of patients undergoing surgery report only partial recovery (e.g., <50% improvement) (1, 9). In such a setting, the prediction of who is likely to respond favorably to decompressive surgery is key to guide surgeons and manage patients’ expectations. There is an urgent need to better understand the pathophysiological mechanisms leading to persistent (and worsening) clinical symptoms associated with DCM, which could ultimately improve the assessment and management of this condition.

Neck pain is increasingly recognized as a key clinical issue in patients with DCM and is associated with perceptions of post-operative quality of life (10). While patients with chronic neck pain demonstrate alterations in cervical muscle morphology (11, 12) and delayed activation during postural perturbations (13), few studies have specifically examined how the cervical muscles may play a role in the development of symptoms and functional impairments in DCM (8, 14). A recent innovation (8) established an association between cervical muscle morphology, clinical symptoms, and functional status in patients with DCM. The same study also reported an increase in multifidus (MF) muscle fatty infiltration at the level below the most cranial level of spinal cord compression, which is most likely related to denervation. A subsequent investigation (14) reported a strong positive correlation between cervical muscle strength and lean muscle mass measured by magnetic resonance imaging (MRI). Furthermore, recent evidence suggested cervical paraspinal muscle morphology and fatty infiltration are predictors of post-surgical outcomes in patients with adjacent segment degeneration undergoing anterior cervical discectomy and fusion (ACDF) (15) as well as in patients undergoing posterior cervical fusion (PCF) (16). Given these findings, it is probable that such variations in cervical muscle morphology and function may contribute to the variability in the surgical outcomes observed in patients with DCM. Therefore, the purpose of this study was to examine whether preoperative cervical muscle size, composition, and asymmetry are predictors of prognosis and functional recovery following surgical treatment in patients with DCM. We hypothesized that smaller cervical muscle, greater pre-surgical asymmetry, and fatty infiltration on clinically warranted MRI scans will be associated with greater symptom severity and lower functional scores post-surgery.

## 2. Materials and methods

### 2.1. Participants

**Figure 1:**
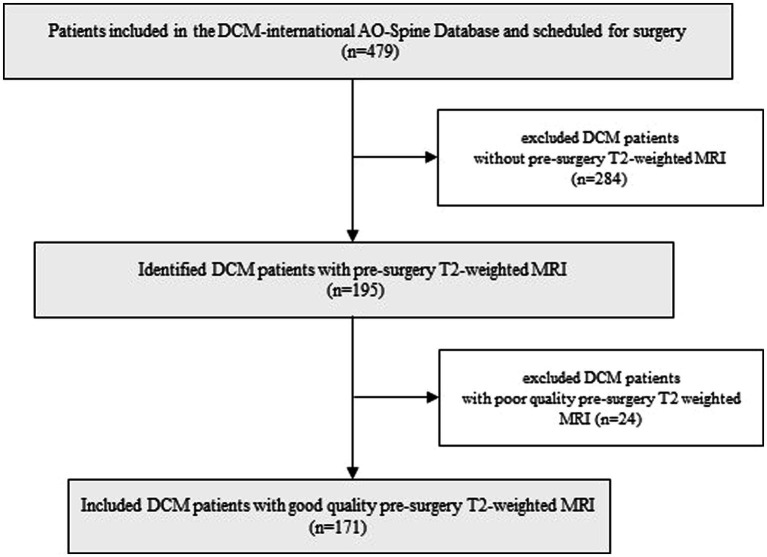
Flowchart depicting the stages and processes involved in including patients.

Patients included in this study were selected from the multicentric Controlled Prospective AOSpine DCM-International cohort study database, which includes a total of 16 different international sites. Of the 479 symptomatic DCM patients comprised in this database and scheduled for surgical treatment, a total of 171 patients were included in the current study. The inclusion criteria included those as follows: (1) good quality pre-surgery MR T2-weighted axial images, (2) aged 18 years or older, (3) presenting with symptomatic DCM with at least one clinical sign of myelopathy, and (4) no previous cervical spine surgery. Patients were excluded if they were asymptomatic or diagnosed with active infection, neoplastic disease, rheumatoid arthritis, ankylosing spondylitis, or concomitant lumbar stenosis (Figure 1). All patients were followed for 2 years, and clinical outcomes were obtained at 6, 12, and 24 months following surgical treatment. Written informed consent was obtained from all patients acknowledging that their data would be used to improve the understanding of DCM. The Controlled Prospective AOSpine DCM-International study was approved by research ethics boards at each center. The Research Ethics Board at University Health Network (Toronto) approved the study at the principal coordinating site (Toronto Western Hospital: PI Michael Fehlings). The Ethics Research Board of McGill University also approved this study (#14-085-GEN).

### 2.2. Procedure

#### 2.2.1. Cervical muscle measurements

**Figure 2:**
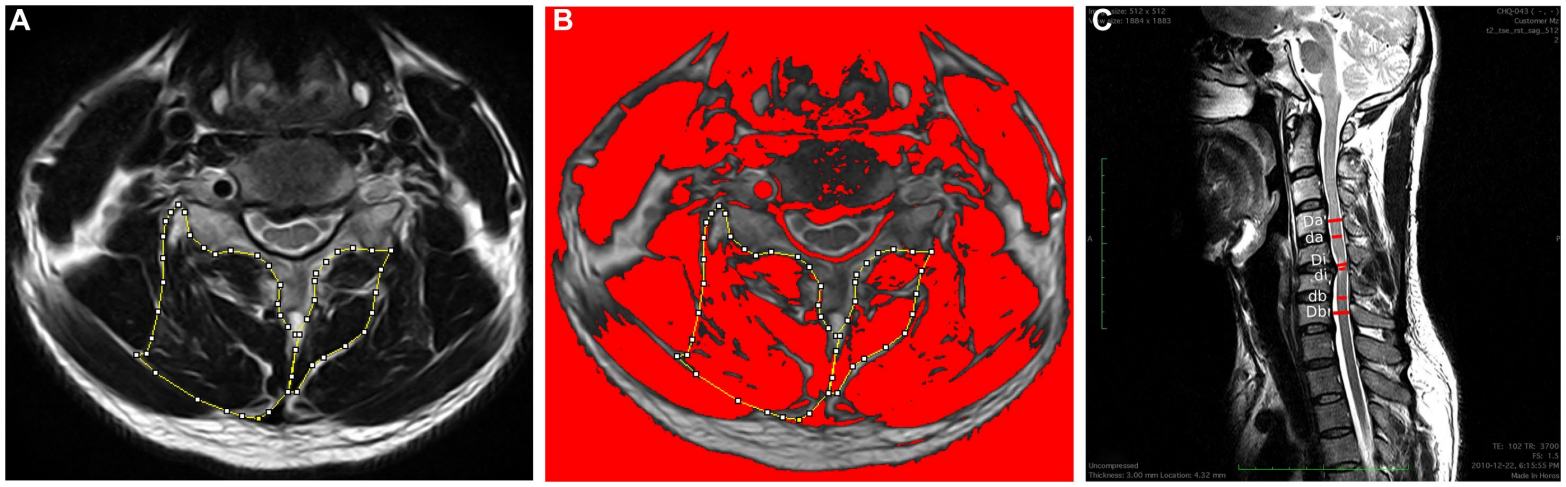
**(A)** Measurements of the total CSA of the MF + Scer muscles and extensor muscles group on axial T2-weighted images at the C4-C5 level. **(B)** The image shows the application of a signal threshold filter (ImageJ) to highlight the fat-free muscle area and obtain the FCSA muscle measurements. **(C)** Measurements required for MCC and MSCC calculation. Di, Da, and Db measure the diameter of the spinal canal at the site of maximum compression and at the nearest normal site above and below, respectively; Di, Da, and Db indicate the diameter of the spinal cord at the site of compression and at the normal site above and below, respectively.

Bilateral cervical muscle measurements included total CSA, functional CSA (FCSA), ratio of FCSA/CSA (fatty infiltration), and asymmetry of the multifidus and semispinalis cervicis (MF + SCer) together and the deep extensor muscles as a group were acquired at the level of maximum cord compression and level below at the mid-disk (Figures 2A,B). The cervical muscle measurements were described in detail elsewhere (17). The following formula defined by Fehlings et al. (18) was used to determine the level and degree of the maximum spinal cord compression (MSCC) and maximum canal compromise (MCC): MSCC = [1 − di (da + db)/2] × 100, and MCC = [1 − Di (Da + Db)/2] × 100 (Figure 2C). The FCSA was measured using a highly reliable thresholding technique described in detail elsewhere (19). The relative percent asymmetry of the paraspinal muscles on an axial view was calculated as follows: the relative asymmetry rate = [(L − S)/L] × 100, where L is the larger side and S is the smaller side (17). To adjust for inter-individual anthropometric differences, total CSA was divided by the size of the disk at the level of interest and relative CSA (RCSA) was used in the analysis. The mean value of the sum of the muscle CSAs or FCSAs on the right and left side at each level and the means for the FCSA/CSA ratio were calculated for each level of interest (e.g., level of max compression, and level below, as well as both levels combined) and used in the analysis.

#### 2.2.2. Self-reported questionnaires

Clinical signs of myelopathy and cervical functional test scores were collected at the time of recruitment (baseline) and followed by clinical and functional scores at 6, 12, and 24 months after surgical treatment. These were used to assess prognosis and functional recovery post-surgery at each time point: modified Japanese Orthopedic Association (mJOA), Nurick Classification, Neck Disability Index (NDI), and SF-36 health survey. The mJOA is an 18-point scale that quantitatively assesses upper and lower extremity motor and sensory function, which has been previously validated (20, 21); however, an additional study revealed that the inter-rater reliability is lower for the upper extremity sensory subscore (ICC = 0.63) (22). The NDI is a self-reported questionnaire used to measure related pain and disability; higher scores (out of 100) are indicative of greater disability. This questionnaire has previously demonstrated good levels of reliability and validity for neck pain (23, 24). The Nurick grade is another objective assessment of the severity of myelopathy but is more heavily weighted on the lower limb function. The score ranges from 0 (lowest disability) to 6 (greatest disability). This metric has been shown to be both reliable and valid regarding functional disability in patients with DCM (3, 25). The SF-36 health survey is a reliable and valid questionnaire, consisting of eight classified scores to measure health-related quality of life. Both physical and mental components of health are assessed in SF-36 health survey. The scores of all questions are summed together to calculate the final score, which is between 0 and 100, with a higher score reflecting a better quality of life (26, 27).

### 2.3. Statistical analysis

Means and standard deviations were calculated for the cervical paraspinal muscle measurements of interest. Univariate and multivariate linear regression analyses were used to assess the relationship between cervical muscle measurements of interest (e.g., independent variables) with post-surgical clinical symptoms and functional outcomes (e.g., dependent variables). Predictors with a univariate value of p of <0.20 were candidates for the multivariable analysis models. Only predictors with a value of p of <0.05 were considered to be statistically significant and retained in the multivariable analysis models. Age, BMI, and sex were considered as possible covariates. Separate models were performed for each level and each clinical outcome at every follow-up time point (e.g., 6-month and 12-month post-surgery). Diagnostic plots were used to assess model assumptions, and all assumptions were found to be tenable. All data analyses were performed with IBM SPSS (version 28.0).

## 3. Results

The average age of the subjects was 54.92 ± 11.85 years (range 28–87), and 112 (65.5%) were men (Table 1). Patients’ characteristics, clinical signs and symptoms, and functional scores are presented in Table 1, and cervical muscle MRI measurements of interest are presented in Table 2. The mean value of the paraspinal muscle measurements of interest at both levels of maximum compression and level below was used as a value of combined level (Table 2).

**Table 1:** Demographic characteristics of patients (*n* = 171).

Characteristics of patients	Mean (SD) or frequency (%)		
Age (year)	54.92 (11.85)		
BMI (kg/m^2^)	25.77 (5.43)		
Sex Male Female	112 (65.5%) 59 (34.5%)		
DCM duration (month)	30.23 (39.63)		
C3–C4 (max level of compression)	39 (22.8%)		
C4–C5 (max level of compression)	48 (28.07%)		
C5–C6 (max level of compression)	68 (39.76%)		
C6–C7 (max level of compression)	16 (9.35%)		
DCM symptoms			
Numb hands	89.8%		
Clumsy hands	71.7%		
Impairment of gait	80.7%		
Bilateral arm paresthesia	57.2%		
L’Hermitte’s phenomena	19.9%		
Weakness	79.5%		
DCM signs			
Corticospinal distribution motor deficits	73.5%		
Atrophy of hand intrinsic muscles	36.1%		
Hyperreflexia	84.3%		
Positive Hoffman sign	66.9%		
Upgoing plantar responses	51.2%		
Lower limb spasticity	64.5%		
Broad-based unstable gait	65.7%		
DCM sources of stenosis			
Spondylosis	84.3%		
Disk	73.49%		
Ossified posterior longitudinal ligament	33.7%		
Hypertrophic ligamentum flavum	33.7%		
Subluxation	5.4%		
Other	0%		
Functional scores	Baseline	6 months	12 months
mJOA	12.05 (2.71)	14.24 (2.56)	14.7 (2.66)
NDI	39.31 (19.28)	26.89 (17.51)	24.59 (18.9)
SF-36	36.84 (12.13)	42.18 (11.32)	42.81 (12.09)
Nurick	3.45(1.21)	2.23 (1.54)	2.13 (1.52)

mJOA, modified Japanese orthopedic association; NDI, neck disability index; SF-36, short-form 36 health survey questionnaire; DCM, degenerative cervical myelopathy; BMI, body mass index; SD, standard deviation.

**Table 2:** Mean (standard deviation) of cervical paraspinal muscle measurements at the level of maximum compression, level below and both combined levels.

Paraspinal muscle measurements		Max level	Level below	Both levels combined
MF + SCer	RCSA	1.16 (0.37)	1.2 (0.34)	1.2 (0.3)
	FCSA/CSA	0.6 (0.16)	0.6 (0.11)	0.6 (0.13)
	CSA asy	10.48 (8.33)	9 (6.97)	9.84 (6.26)
	FCSA asy	13.31 (11.37)	13.13 (10.25)	13.26 (8.49)
	FCSA/CSA asy	11.07 (9.95)	11.09 (9.03)	11.2 (7.5)
Muscle group	RCSA	3.21 (1.1)	2.85 (0.9)	3.03 (0.92)
	FCSA/CSA	0.68 (0.09)	0.69 (0.09)	0.68 (0.07)
	CSA asy	7.16 (6.36)	6.65 (5.17)	6.83 (4.41)
	FCSA asy	7.6 (7.5)	7.21 (6.34)	7.32 (5)
	FCSA/CSA asv	5.8 (5.06)	6.52 (5.44)	6.2 (3.99)

CSA, cross-sectional area; FCSA, functional cross-sectional area; MF, multifidus muscle; SCer, semispinalis cervicis; Asy, asymmetry; RCSA, relative cross-sectional area.

### 3.1. Association between preoperative muscle parameters and functional scores at 6-month post-surgery

Univariate and multivariate regression analyses for cervical muscle parameters of interest and covariates (age, sex, gender, and BMI) with mJOA at 6-month post-surgery are presented in Table 3. FCSA/CSA MF + SCer, FCSA/CSA of the muscle group, CSA asymmetry of MF + SCer at both below and combined levels, RCSA for the muscle group at the level of maximum compression and combined level, and RCSA of the MF + SCer at the level of most compression and age were associated with mJOA in the univariate analysis and entered the multivariable model. Lower RCSA of the muscle group at the level of maximum compression (value of *p* = 0.034), less CSA MF + SCer asymmetry (value of p = <0.001), and greater FCSA/CSA of the muscle group (e.g., less fatty infiltration) (value of *p* = 0.004) at the level below and younger age (value of *p* = 0.024) were significant predictors of higher mJOA scores (e.g., less disability) at 6-month post-surgery (Table 3).

**Table 3:** Results of univariate and multivariate regression analyses and mJOA after 6-month post-surgery.

Paraspinal muscle measurements		Univariate analysis(Coeff) [95% CI]	*p*-value	Multivariate analysis (Coeff) [95% CI]	*p*-value
Max level					
MF + SCer	RCSA	−0.7 [−1.758, 0.358]	0.193		
	FCSA/CSA	0.729 [−1.673, 3.132]	0.55		
	CSA asy	−0.024 [−0.071, 0.023]	0.32		
	FCSA asy	−0.018 [−0.053, 0.016]	0.301		
	FCSA/CSA asy	−0.006 [−0.046, 0.033]	0.745		
Muscle group	RCSA	−0.272 [−0.631, 0.088]	0.137	−0.158 [−0.710, −0.028]	0.034*
	FCSA/CSA	0.674 [−3.357, 4.704]	0.742		
	CSA asy	0.025 [−0.037, 0.086]	0.427		
	FCSA asy	0.021 [−0.032, 0.073]	0.44		
	FCSA/CSA asy	0.021 [−0.056, 0.099]	0.585		
Level below					
MF + SCer	RCSA	−0.457 [−1.618, 0.704]	0.438		
	FCSA/CSA	5.159 [1.685, 8.632]	0.004*		
	CSA asy	−0.081 [−0.136, −0.026]	0.004*	−0.249 [−0.144, −0.038]	<0.001*
	FCSA asy	−0.004 [−0.042, 0.035]	0.84		
	FCSA/CSA asy	−3.034E-5 [−0.044, 0.044]	0.999		
Muscle group	RCSA	−0.206 [−0.647, 0.235]	0.358		
	FCSA/CSA	6.749 [2.412, 11.086]	0.002*	0.211 [1.921, 10.269]	0.004*
	CSA asy	−0.046 [−0.122, 0.03]	0.233		
	FCSA asy	−0.035 [−0.097, 0.026]	0.259		
	FCSA/CSA asy	0.007 [−0.065, 0.079]	0.849		
Both levels combined					
MF + SCer	RCSA	−0.838 [−2.156, 0.479]	0.211		
	FCSA/CSA	3.218 [−0.224, 6.661]	0.067		
	CSA asy	−0.073 [−0.135, −0.010]	0.023*		
	FCSA asy	−0.02 [−0.066, 0.027]	0.411		
	FCSA/CSA asy	−0.006 [−0.059, 0.047]	0.826		
Muscle group	RCSA	−0.291 [−0.720, 0.138]	0.182		
	FCSA/CSA	4.778 [−0.178, 9.733]	0.059		
	CSA asy	−0.005 [−0.095, 0.084]	0.905		
	FCSA asy	−0.005 [−0.083, 0.072]	0.891		
	FCSA/CSA asy	0.025 [−0.075, 0.124]	0.629		
Patients’ characteristics					
Age		−0.032 [−0.065, 0.001]	0.057	−0.168 [−0.068, −0.005]	0.024*
Gender		−0.501 [−1.353, 0.350]	0.247		
BMI		0.001 [−0.075, 0.077]	0.985		
DCM duration		−0.001 [−0.011, 0.009]	0.834		

CSA, cross-sectional area; FCSA, functional cross-sectional area; RCSA, ratio cross-sectional area; MF, multifidus muscle; SCer, semispinalis cervicis; Asy, asymmetry; mJOA, modified Japanese orthopedic association; BMI, body mass index; Coeff, coefficient; CI, confidence interval; DCM, degenerative cervical myelopathy, *p < 0.05.

Univariate and multivariate regression analyses for Nurick scores at 6-month post-surgery are presented in Table 4. FCSA/CSA MF + SCer, CSA MF + SCer asymmetry at the level of maximum compression, level below and combined level, FCSA/CSA of the muscle group at both, the below and combined level, RCSA of the MF + SCer, FCSA asymmetry of the MF + SCer, and RCSA at the level of maximum compression and FCSA asymmetry of the muscle group at the level below were all associated with the Nurick score in the univariate analysis and entered the multivariable model. Less FCSA/CSA of the muscle group (e.g., greater fatty infiltration; value of *p* = 0.002) at the level below and greater CSA asymmetry MF + SCer (value of *p* = 0.018) at the combined level remained significant predictors of a higher Nurick score at 6-month post-surgery in the multivariable model. Lower FCSA asymmetry of MF + SCer was also associated with higher NDI scores at 6-month post-surgery. Finally, greater asymmetry in FCSA/CSA of the MF + SCer at the level of maximum compression and greater FCSA asymmetry of the muscle group at the level below were correlated with lower post-operative SF-36 scores (*p* = 0.045 and 0.018, respectively) in the multivariable model.

**Table 4:** Results of univariate and multivariate regression analyses and Nurick after 6-month post-surgery.

Paraspinal muscle measurements		Univariate analysis (Coeff) [95% CI]	*p*-value	Multivariate analysis (Coeff) [95% CI]	*p*-value
Max level					
MF + SCer	RCSA	0.419 [−0.218, 1.056]	0.196		
	FCSA/CSA	−1.048 [−2.486, 0.391]	0.152		
	CSA asy	0.022 [−0.006, 0.051]	0.123		
	FCSA asy	0.015 [−0.005, 0.036]	0.146		
	FCSA/CSA asy	0.01 [−0.014, 0.034]	0.4		
Muscle group	RCSA	0.152 [−0.064, 0.369]	0.167		
	FCSA/CSA	−0.26 [−2.686, 2.167]	0.833		
	CSA asy	−0.008 [−0.045, 0.029]	0.672		
	FCSA asy	0.008 [−0.023, 0.04]	0.6		
	FCSA/CSA asy	0.005 [−0.042, 0.051]	0.84		
Level below					
MF + SCer	RCSA	0.053 [−0.647, 0.753]	0.882		
	FCSA/CSA	−3.017 [−5.111, −0.923]	0.005*		
	CSA asy	0.036 [0.003, 0.07]	0.034*		
	FCSA asy	0.004 [−0.019, 0.028]	0.702		
	FCSA/CSA asy	0.005 [−0.022, 0.031]	0.727		
Muscle group	RCSA	0.014 [−0.252, 0.28]	0.916		
	FCSA/CSA	−3.940 [−6.555, −1.325]	0.003*	−0.232 [−6.606, −1.447]	0.002*
	CSA asy	0.023 [−0.023, 0.069]	0.319		
	FCSA asy	0.027 [−0.011, 0.064]	0.159		
	FCSA/CSA asy	0.004 [−0.039, 0.048]	0.854		
Both levels combined					
MF + SCer	RCSA	0.359 [−0.436, 1.154]	0.374		
	FCSA/CSA	−2.532 [−4.589, −0.475]	0.016*		
	CSA asy	0.043 [0.005, 0.08]	0.026*	0.180 [0.008, 0.081]	0.018*
	FCSA asy	0.017 [−0.011, 0.045]	0.223		
	FCSA/CSA asy	0.013 [−0.019, 0.045]	0.434		
Muscle group	RCSA	0.115 [−0.144, 0.374]	0.382		
	FCSA/CSA	−2.692 [−5.686, 0.302]	0.078		
	CSA asy	0.007 [−0.046, 0.061]	0.784		
	FCSA asy	0.03 [−0.016, 0.077]	0.203		
	FCSA/CSA asy	0.008 [−0.052, 0.068]	0.796		
Patients’ characteristics					
Age		0.008 [−0.012, 0.029]	0.407		
Gender		0.196 [−0.315, 0.707]	0.45		
BMI		−0.008 [−0.054, 0.038]	0.731		
DCM duration		−0.003 [−0.009, 0.003]	0.39		

CSA, cross-sectional area; FCSA, functional cross-sectional area; RCSA, ratio cross-sectional area; MF, multifidus muscle; SCer, semispinalis cervicis; Asy, asymmetry; mJOA, modified Japanese orthopedic association; BMI, body mass index; Coeff, coefficient; CI, confidence interval; DCM, degenerative cervical myelopathy, *p < 0.05.

### 3.2. Association between preoperative muscle parameters and functional scores at 12-month post-surgery

Univariate and multivariate regression analyses for mJOA scores at 12-month post-surgery are presented in Table 5. Lower RCSA of both the MF + Scer and muscle group at all levels was associated with higher mJOA scores (e.g., lower disability) in the univariate analysis. Greater FCSA/CSA (e.g., less fatty infiltration) of the MF + SCer and muscle group at the level below and combined level and lower CSA asymmetry of the MF + SCer at the level below were all significantly associated with higher mJOA scores at 12-month post-surgery in the univariate analysis. Lower CSA asymmetry of MF + SCer (*p* = 0.005), greater FCSA/CSA of the muscle group at the level below (*p* = 0.002), and lower CSA asymmetry of the muscle group at both levels combined and younger age (*p* = 0.032) were significant predictors of higher mJOA (e.g., less disability) scores at 12-month post-surgery in the multivariable model.

**Table 5:** Results of univariate and multivariate regression analyses and mJOA after 12-month post-surgery.

Paraspinal muscle measurements		Univariate analysis (Coeff) [95% CI]	*p*-value	Multivariate analysis (Coeff) [95% CI]	*p*-value
Max level					
MF + SCer	RCSA	−1.236 [−2.323, −0.148]	0.026 *		
	FCSA/CSA	1.12 [−1.371, 3.61]	0.376		
	CSA asy	−0.002 [−0.052, 0.047]	0.929		
	FCSA asy	−0.004 [−0.04, 0.032]	0.82		
	FCSA/CSA asy	−0.005 [−0.046, 0.036]	0.799		
Muscle group	RCSA	−0.5 [−0.869, −0.13]	0.008*		
	FCSA/CSA	1.363 [−2.822, 5.547]	0.521		
	CSA asy	0.06 [−0.003, 0.123]	0.063		
	FCSA asy	0.023 [−0.032, 0.078]	0.407		
	FCSA/CSA asy	0.033 [−0.048, 0.113]	0.427		
Level below					
MF + SCer	RCSA	−1.493 [−2.68, −0.307]	0.014*		
	FCSA/CSA	5.396 [1.776, 9.015]	0.004*		
	CSA asy	−0.063 [−0.121, −0.005]	0.033*	−0.212 [−0.135, −0.025]	0.005*
	FCSA asy	−0.008 [−0.048, 0.032]	0.705		
	FCSA/CSA asy	0.009 [−0.036, 0.055]	0.684		
Muscle group	RCSA	−0.601 [−1.053, −0.149]	0.010*		
	FCSA/CSA	7.417 [2.922, 11.913]	0.001*	0.231 [2.583, 11.139]	0.002*
	CSA asy	−0.004 [−0.089, 0.08]	0.92		
	FCSA asy	−0.048 [−0.115, 0.02]	0.164		
	FCSA/CSA asy	0.004 [−0.072, 0.081]	0.915		
Both levels combined					
MF + SCer	RCSA	−1.922 [−3.264, −0.581]	0.005*		
	FCSA/CSA	3.722 [0.156, 7.288]	0.041*		
	CSA asy	−0.042 [−0.108, 0.024]	0.211		
	FCSA asy	−0.01 [−0.059, 0.039]	0.7		
	FCSA/CSA asy	0.002 [−0.053, 0.058]	0.939		
Muscle group	RCSA	−0.64 [−1.078, −0.201]	0.005*		
	FCSA/CSA	5.711 [0.584, 10.838]	0.029*		
	CSA asy	0.063 [−0.031, 0.157]	0.189	−0.265 [−1.181, −0.343]	<0.001*
	FCSA asy	−0.009 [−0.092, 0.074]	0.826		
	FCSA/CSA asy	0.031[−0.073, 0.135]	0.559		
Patients’ characteristics					
Age		−0.03 [−0.065, 0.005]	0.088	−0.159 [−0.068, −0.003]	0.032*
Gender		−0.172 [−1.06, 0.716]	0.702		
BMI		0.03 [−0.049, 0.11]	0.456		
DCM duration		0.0 [−0.011, 0.01]	0.974		

CSA, cross-sectional area; FCSA, functional cross-sectional area; RCSA, ratio cross-sectional area; MF, multifidus muscle; SCer, semispinalis cervicis; Asy, asymmetry; mJOA, modified Japanese orthopedic association; BMI, body mass index; Coeff, coefficient; CI, confidence interval; DCM, degenerative cervical myelopathy, *p < 0.05.

Univariate and multivariate regression analyses with Nurick scores at 12-month post-surgery are presented in Table 6. Greater RCSA for MF + Scer and muscle group at almost all levels, lower FCSA/CSA for the MF + SCer at the level below and combined levels, and greater MF + Scer CSA asymmetry at the level below and muscle group FCSA/CSA asymmetry (combined levels) were all significantly associated with higher Nurick scores (e.g., more disability) at 12-month post-surgery in the univariate analyses. However, only greater RCSA for the muscle group at the maximum level and greater asymmetry for the MF + Scer at the level below and lower FCSA/CSA (e.g., more fatty infiltration) for the muscle group at the level below remained significant in the multivariable model.

**Table 6:** Results of univariate and multivariate regression analyses and Nurick after 12 months following surgery.

Paraspinal muscle measurements		Univariate analysis (Coeff) [95% CI]	*p*-value	Multivariate analysis (Coeff) [95% CI]	*p*-value
Max level					
MF + SCer	RCSA	0.942 [0.328, 1.556]	0.003*		
	FCSA/CSA	−1.285 [−2.698, 0.128]	0.074		
	CSA asy	0.011 [−0.018, 0.039]	0.456		
	FCSA asy	0.009 [−0.012, 0.029]	0.407		
	FCSA/CSA asy	0.002 [−0.021,0.025]	0.872		
Muscle group	RCSA	0.295 [0.084, 0.505]	0.006*	0.235 [0.126, 0.525]	0.002*
	FCSA/CSA	−1.584 [−3.966, 0.798]	0.191		
	CSA asy	−0.014 [−0.05, 0.023]	0.463		
	FCSA asy	0.005 [−0.026, 0.036]	0.743		
	FCSA/CSA asy	−0.001 [−0.047, 0.045]	0.958		
Level below					
MF + SCer	RCSA	0.696 [0.014, 1.379]	0.046*		
	FCSA/CSA	−3.058 [−5.128, −0.988]	0.004*		
	CSA asy	0.042 [0.008, 0.075]	0.014*	0.211 [0 0.014, 0.077]	0.005*
	FCSA asy	5.043E-5[−0.023,0.023]	0.997		
	FCSA/CSA asy	−0.006 [−0.032, 0.02]	0.665		
Muscle group	RCSA	0.26 [−0.001, 0.521]	0.051		
	FCSA/CSA	−4.734 [−7.283, −2.185]	<0.001*	−0.270 [−7.035, −2.154]	<0.001*
	CSA asy	0.008 [−0.041, 0.056]	0.757		
	FCSA asy	0.014 [−0.025, 0.053]	0.469		
	FCSA/CSA asy	0.007 [−0.037, 0.05]	0.766		
Both levels combined					
MF + SCer	RCSA	1.179 [0.416, 1.943]	0.003*		
	FCSA/CSA	−2.79 [−4.809, −0.772]	0.007*		
	CSA asy	0.036 [−0.002, 0.073]	0.061		
	FCSA asy	0.008 [−0.02, 0.036]	0.573		
	FCSA/CSA asy	−0.002 [−0.034, 0.029]	0.877		
Muscle group	RCSA	0.333 [0.081, 0.584]	0.01*		
	FCSA/CSA	−4.207 [−7.115, −1.299]	0.005*		
	CSA asy	−0.01 [−0.064, 0.044]	0.712		
	FCSA asy	0.017 [−0.031, 0.064]	0.489		
	FCSA/CSA asy	0.005 [−0.054, 0.065]	0.866		
Patients’ characteristics					
Age		0.004 [−0.016, 0.024]	0.675		
Gender		0.119 [−0.387, 0.626]	0.642		
BMI		−0.009 [−0.055, 0.036]	0.685		
DCM duration		0.002 [−0.003, 0.008]	0.412		

CSA, cross-sectional area; FCSA, functional cross-sectional area; RCSA, ratio cross-sectional area; MF, multifidus muscle; SCer, semispinalis cervicis; Asy, asymmetry; mJOA, modified Japanese orthopedic association; BMI, body mass index; Coeff, coefficient; CI, confidence interval; DCM, degenerative cervical myelopathy, *p < 0.05.

Our results demonstrated that RCSA of the MF + SCer, FCSA asymmetry of MF + Scer, and FCSA/CSA asymmetry of the muscle group at all measured levels were associated with NDI in univariate analysis. Lower FCSA/CSA asymmetry of the muscle group (value of *p* = 0.050) and greater RCSA MF + SCer (value of *p* = 0.034) measured of the combined level remained significant in the multiple regression analysis with a higher NDI score at 12 weeks post-surgery. RCSA of the muscle group at the below level (value of *p* = 0.003) and CSA asymmetry of MF + SCer at the combined level (value of *p* = 0.042) had a negative significant relationship with SF-36 post-surgery in the multivariable analysis (results not presented).

## 4. Discussion

Our analysis revealed that several cervical muscle morphology characteristics were predictors of improved mJOA scores (indicating less disability) at 6 and 12 months after surgery, adding importance to the identification of preoperative factors that could potentially be optimized before surgery to enhance recovery after surgery (ERAS) (28). Our findings provide more evidence that clinical and imaging features of muscle composition and morphology can play a role in classifying those who will benefit from surgery (29–31) and should be considered for selecting patients that would be suitable for ERAS pathways versus those that might require a more extensive in-hospital stay after surgery (32).

Smaller deep cervical extensors muscle size (e.g., reduced RCSA of the muscle group) at the maximum level of compression, less asymmetry in the CSA of MF + SCer, and greater FCSA/CSA for the group of muscles (indicating less fatty infiltration) below the maximum level of compression and less asymmetry of the muscle group at both combined levels were all associated with better post-surgery outcomes at both 6 and 12 months after surgery. The fact that reduced CSA is associated with better outcomes may be related to our measurement protocol. As we only assessed MF + Scer and the entire cervical extensor group, interstitial fat, if present, was included in the region of interest (ROI), which may have influenced our results. This hypothesis is further supported by the fact that we also found an association between greater muscle fat (lower FCSA/CSA) and worse post-operative outcomes. In addition, younger age was also a significant predictor of improved mJOA scores (all *p* < 0.05). Greater CSA asymmetry in MF + SCer and lower FCSA/CSA (e.g., more fatty infiltration) for the cervical muscle group at the below level of compression and greater RCSA of the cervical muscle group at most compression level were significant predictors of higher Nurick scores (e.g., more disability) at both 6-month and 12-month post-surgery.

Therefore, muscle parameters, such as fatty infiltration and asymmetry, may have an impact on the prognosis and functional recovery of patients with DCM (8). Our results, suggesting an association between cervical muscle fat infiltration and clinical outcomes (e.g., mJOA score and Nurick scores), are in line with prior research in DCM and whiplash-associated disorders (11, 12). Patients with whiplash-associated disorders who nominated self-recovery at 12-month post-injury had significantly less neck muscle fat infiltration in the multifidus muscle (33). The presence of greater fatty infiltration and asymmetry in these muscles may be associated with worse functional scores, clinical signs, and symptoms (8, 14, 33).

Previous research reported that fatty infiltration of the semispinalis capitis (SCap) was linked to mJOA scores in DCM patients (8). In contrast, Cloney et al. (1) revealed that increased muscle fat infiltration of MF + Scer was correlated with decreased sensorimotor function as measured by the mJOA and Nurick scores, while Fortin et al. (8) reported no relationship between MF fat infiltration and mJOA scores. However, since both muscles are deep extensors that play a significant role in the stability of the cervical spine, their pathologies are probably reflected in overlapping clinical manifestations that are quantified by the mJOA score (1). Alternately, various other factors, including the level of measurement selected, might have had an impact on the findings and measurements of paraspinal muscles as Fortin et al. (8) only included symptomatic DCM patients with the most level of compression at C4-C5 and C5-C6 levels. Furthermore, in the current study and Cloney’s study, MF and Scer were segmented together (e.g., same ROI) as the boundary between these two muscles is not always clearly visible at all levels, while Fortin et al. (8) measured the MF by itself. In another study, however, Fortin et al. (14) observed an association between a greater mean FCSA/CSA ratio of the entire cervical extensor group (e.g., less fatty infiltration) with a higher mJOA score (e.g., lower disability). Similar to the current study, cervical muscle measurements were obtained bilaterally at the mid-disk from C2 to C7. In the lumbar spine, evidence clearly suggests that lower paraspinal muscle quality is associated with decreased strength, increased frailty, increased risks of fractures and falls, and worst post-operative outcomes (34–36). In addition to establishing the significance of preoperative muscle morphometry in predicting outcomes in DCM, our study has identified two novel predictors (deep extensor fat infiltration and asymmetry) of functional recovery after surgery. These findings demonstrate that deep extensor sarcopenia can likely be used as a predictive factor for poor Nurick grade and mJOA improvement post-surgery.

The effect of age on surgical outcomes in patients with DCM has been a topic of debate and research (4, 9, 37). Some studies suggest that younger age is a significant predictor of better outcomes, while others report that age is not a clear predictor (9, 37–39). Zileli et al. (37) found that age was a significant factor influencing outcomes in DCM patients, but no specific age cutoff value could predict the outcome. Tetreault et al. (38) hypothesized that reduced physiological reserves, poorer overall health status, and increased comorbidities may make older patients more susceptible to complications following DCM surgery. They found that age was a significant predictor of complications in their study. Overall, the effect of age on DCM surgical outcomes remains complex and requires further investigation.

While lower FCSA asymmetry of MF + Scer was associated with higher NDI scores at 6-month post-surgery, lower FCSA/CSA asymmetry of group muscle and greater RCSA MF + Scer were associated with higher NDI scores at 12-month post-surgery. This result is consistent with our previous study that has been recently published suggesting an association between lower asymmetry in cervical muscle morphology and increased NDI scores in baseline measurements (17). In contrast, Fortin et al. (8) reported an association between higher NDI scores and greater asymmetry in fatty infiltration of the semispinalis capitis muscle in patients with DCM. In the current study, however, the semispinalis capitis was not assessed individually but was included as part of the muscle group ROI, which may explain the different results. Furthermore, our study investigated the relationship between preoperative muscle morphology measurements and post-surgical outcome, while Fortin’s study assessed the relationship between preoperative muscle characteristics and preoperative clinical outcomes. Lastly, Fortin et al. only included patients with spinal cord compression at C4-C5 and C5-C6 as their first level compression (e.g., most caudal level of compression). In the current study, all the levels were considered (e.g., from C2 to C7), and cervical muscle measurements were obtained in relation to the level of maximal cord compression. Finally, we found lower RCSA and lower FCSA asymmetry of the muscle group and lower asymmetry in FCSA/CSA and CSA of the MF + SCer had a significant relationship with higher SF-36 scores at 6-month and 12-month post-surgery. Fortin et al. reported no association between preoperative cervical muscle characteristics and preoperative SF-36 scores, (14) which is in accordance with our previous study (17). Therefore, SF-36 scores are likely not the best indicator of cervical muscle characteristics in this population.

While there is a growing body of evidence suggesting that surgery has a positive impact on patients with DCM (40), the role of non-operative treatment in this patient population is less clear (41–43). Rehabilitation plays a crucial role in the management of patients with neurological disabilities, including those with DCM and its importance should not be neglected (42, 43). Conservative rehabilitation can help patients with DCM achieve their maximum potential in terms of function and independence, as well as improve their overall wellbeing (42, 44). Our results suggest that exercise therapy including a range of motion and strengthening exercises to improve cervical muscle characteristics could likely enhance patients’ outcomes. It has been demonstrated that timely and strategic rehabilitation is essential for maximizing functional outcomes in other neurological disorders such as stroke; therefore, it is crucial that appropriate perioperative rehabilitative interventions should be implemented, alongside surgical approaches to achieve the best possible outcomes (42, 44).

There are several limitations to our study that should be noted. First, as the paraspinal muscle morphology has been measured in different levels from C2 to C7, MF and Scer were regarded as a single group of muscles and the paraspinal muscle as another one as the precise border between each muscle was not always discernible. Second, we did not consider the impact of pre-surgery conservative treatment on the morphology of the deep extensor neck muscles. Third, T2-weighted images were used in the current study and acquired from different institutions, and therefore, the imaging scanner parameters were not standardized. Furthermore, only MRI assessment of muscle morphology/composition was performed, and additional measures of cervical muscle function should be considered in future study. Additionally, our analyses included numerous comparisons, which raised the possibility of chance finding or type I errors. It is also worth noting that deep learning automatic segmentation methods, such as convolutional neural networks, are advancing and have been used in a clinical population of patients with DCM (45) and whiplash (46) to rapidly and accurately evaluate the cervical muscles.

## 5. Conclusion

Our findings suggest that preoperative cervical muscle morphology/composition, specifically greater asymmetry, and fatty infiltration may be predictors of poor surgical outcomes. In other words, patients who have more severe changes in cervical muscle morphology may be less likely to experience and nominate good functional recovery post-surgery. This highlights the importance of considering muscle parameters in the assessment and treatment of patients with DCM. It would also be beneficial to examine whether variations in paraspinal muscle morphology and composition, as well as functional results, are influenced by changes in cervical lordosis and sagittal parameters (28, 47). Healthcare professionals may need to evaluate cervical muscle function and structure as part of their management plan for these patients to optimize their recovery and improve their outcomes. This study opens the possibility of targeting cervical muscle strengthening in ERAS protocols prior to undertaking surgery for DCM in individuals with compromised cervical muscle morphology (28).

## Data availability statement

The raw data supporting the conclusions of this article will be made available by the authors, without undue reservation.

## Ethics statement

The studies involving humans were approved by The Research Ethics Board at University Health Network (Toronto) approved the study at the principal coordinating site (Toronto Western Hospital: PI MGF). The Ethics Research Board of McGill University also approved this study (#14-085-GEN). The studies were conducted in accordance with the local legislation and institutional requirements. The participants provided their written informed consent to participate in this study.

## Author contributions

NN participated in the research design, acquired the MRI cervical muscle measurements, completed the data analysis, and drafted the manuscript. JME participated in the research design, interpreted the results, and critically reviewed the manuscript. MHW and MGF provided access to the data and critically reviewed the manuscript. MF contributed to the conception, design of the study, data analysis, and interpretation of the data. All authors have read and approved the final manuscript.

## Funding

MF was supported by the Fond de la Recherche en Santé du Québec (FRQS—chercheur boursier Junior 1, grant#283321).

## Conflict of interest

The authors declare that the research was conducted in the absence of any commercial or financial relationships that could be construed as a potential conflict of interest.

## Publisher’s note

All claims expressed in this article are solely those of the authors and do not necessarily represent those of their affiliated organizations, or those of the publisher, the editors and the reviewers. Any product that may be evaluated in this article, or claim that may be made by its manufacturer, is not guaranteed or endorsed by the publisher.
